# Micro-dislodgement during transcatheter aortic valve implantation with a contemporary self-expandable prosthesis

**DOI:** 10.1371/journal.pone.0224815

**Published:** 2019-11-07

**Authors:** Katharina Hellhammer, Kerstin Piayda, Shazia Afzal, Verena Veulemans, Inga Hennig, Matthias Makosch, Amin Polzin, Malte Kelm, Tobias Zeus

**Affiliations:** 1 University Hospital Düsseldorf, Medical Faculty, Department of Cardiology, Pulmonology and Vascular Medicine, Düsseldorf, Germany; 2 CARID (Cardiovascular Research Institute Düsseldorf), Düsseldorf, Germany; Erasmus Medical Center, NETHERLANDS

## Abstract

**Objectives:**

To evaluate the incidence, risk factors and the clinical outcome of micro-dislodgement (MD) with a contemporary self-expandable prosthesis during transcatheter aortic valve implantation.

**Methods:**

MD was defined as movement of the prosthesis of at least 1.5 mm upwards or downwards from its position directly before release compared to its final position. Patients were grouped according to the occurrence (+MD) or absence (-MD) of MD. Baseline characteristics, imaging data and outcome parameters were retrospectively analyzed.

**Results:**

We identified 258 eligible patients. MD occurred in 31.8% (n = 82) of cases with a mean magnitude of 2.8 mm ± 2.2 in relation to the left coronary cusp and 3.0 mm ± 2.1 to the non-coronary cusp. Clinical and hemodynamic outcomes were similar in both groups with consistency over a follow-up period of three months. A larger aortic valve area (AVA) (-MD vs. +MD: 0.6 cm^2^ ± 0.3 vs. 0.7cm^2^ ± 0.2; p = 0.014), was the only independent risk factor for the occurrence of MD in a multivariate regression analysis (OR 5.3; 95% CI: 1.1–24.9; p = 0.036).

**Conclusions:**

MD occurred in nearly one third of patients and did not affect clinical and hemodynamic outcome. A larger AVA seems to be a potential risk factor for MD.

## Introduction

Since 2002, transcatheter aortic valve implantation (TAVI) has emerged to a favorable treatment option for patients with symptomatic severe aortic stenosis (AS) who are deemed inoperable or present with a high surgical risk [1]. Nowadays, this minimal-invasive procedure has become an inherent part of daily cardiovascular care and even qualifies for the treatment of an intermediate risk clientele [2, 3]. Recent studies have focused on TAVI in low-risk patients, which showed a non-inferiority to surgery with regard to the composite endpoint of death or disabling stroke [4] or even a lower rate of the composite of death, stroke or rehospitalization at one year [5] However, due to the limited data on TAVI in low-risk patients and long-term durability data, surgical treatment still remains the first choice in these patients, who are mostly of younger age and present more often with a bicuspid valve anatomy [3]. TAVI undergoes constant technical development which improved patients’ safety and their clinical outcomes over the last years [6–8]. Currently, two device categories are in widespread use: On the one hand self-expandable valves and on the other hand balloon-expandable valves. Both device categories provide excellent clinical results. However, pacemaker rate and incidence of paravalvular leakage (PVL) has been observed to be higher in self-expandable valves [9] which may be due to the lower radial strength of the nitinol frame, an increased angulation between the left ventricular outflow tract (LVOT) and the ascending aorta and a deeper frame position within the LVOT [10]. Balloon-expandable valves are not re-sheathable once they are implanted and carry a higher risk of annulus perforation, especially in highly calcified anatomies [11].

Throughout the implantation, precise positioning of the prosthesis is a crucial step which determines procedural success in particular [12]. If self-expandable prostheses are used, micro-dislodgement (MD) can be observed in some cases during the final phase of valve deployment. Although this is a well-known and often observed phenomenon, data about incidence, risk factors and clinical implication are scarce. Previous studies mostly focused on complete valve embolization, which is a rare complication [13–15]. Micro-dislodgement may affect the hemodynamic results of the valve, especially with regard to PVL, which is a major concern in self-expandable valves. Suboptimal placement of the valve, as for example a too deep implantation, may cause a higher degree of PVL, which is associated with an increased morbidity and mortality [16]. In addition, conduction disturbances may occur more frequent if the valve moves deeper in the LVOT during final release.

In this study, we aimed to establish a definition for MD and evaluated its incidence, potential risk factors and associated clinical outcomes in a real-world TAVI collective.

## Methods

**Fig 1:**
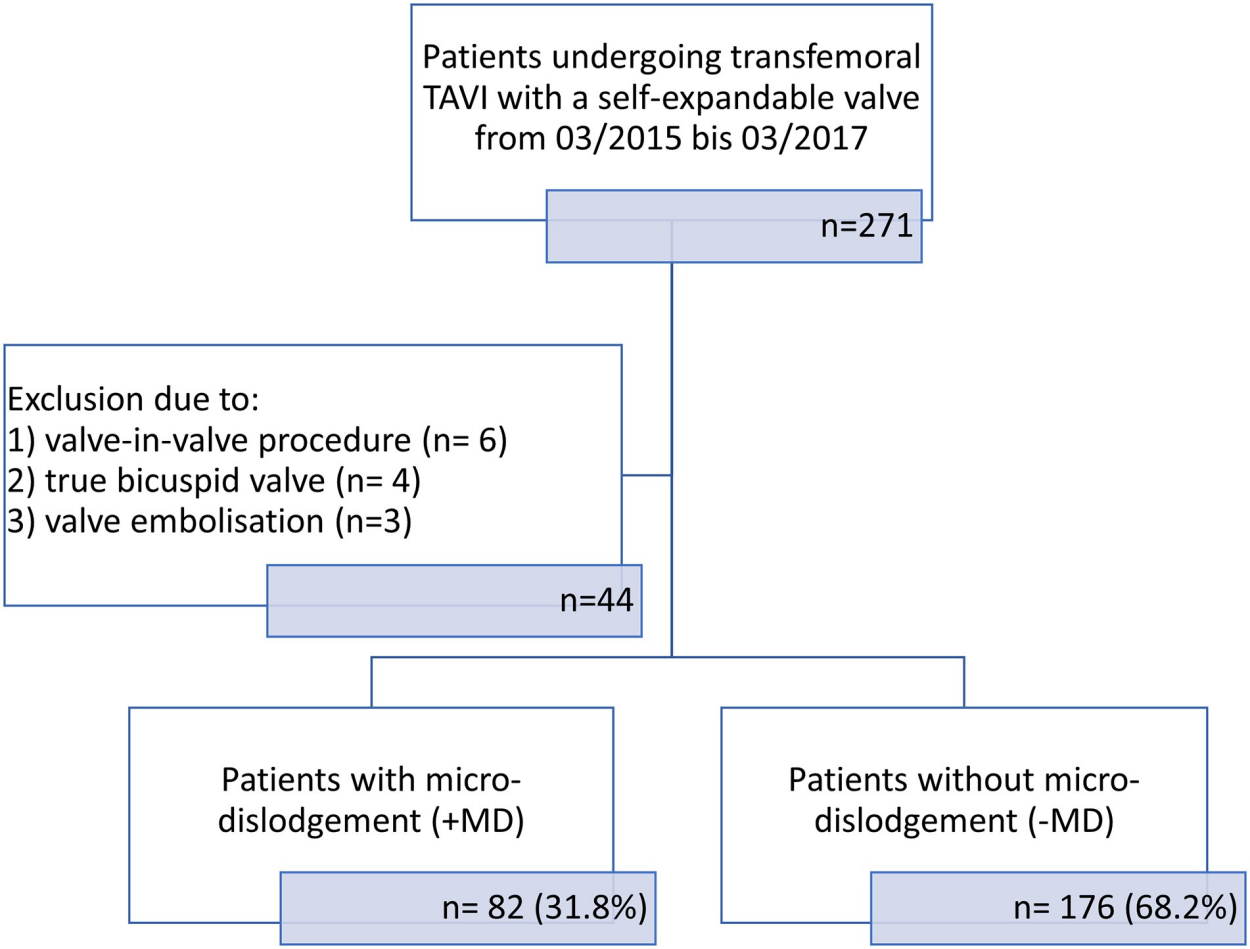
CONSORT Flow diagram. We identified 271 eligible patients who underwent transfemoral transcatheter aortic valve implantation with the Medtronic Corevalve system from 03/2015-03/2017. We excluded valve-in-valve procedures (n = 6), true bicuspid valves (n = 4), and patients with valve embolization (n = 3). Patients were grouped according to the occurrence (+MD) or the absence (-MD) of micro-dislodgement (MD) and further analyzed. TAVI: transcatheter aortic valve implantation.

In a single-center retrospective study we identified 271 consecutive patients who underwent transfemoral TAVI with the self-expandable Medtronic CoreValve system from 03/2015–03/2017. We excluded patients receiving a valve-in-valve procedure (n = 6), true bicuspid aortic valves (n = 4), and patients with valve embolization (n = 3). A CONSORT flow diagram (Fig 1) provides an overview of the patient selection process. Eligible candidates were discussed in our interdisciplinary heart team and considered suitable for TAVI due to severe symptomatic aortic stenosis and high surgical risk or contraindication for a conventional open-heart surgery. All patients provided written informed consent for data acquisition and analysis. The study was approved by the local ethics committee of the Heinrich-Heine University Düsseldorf, is registered as a clinical trial (NCT 01805739) and was performed in accordance to the Declaration of Helsinki. All patients provided written informed consent for data acquisition and analysis.

TAVI procedure was performed under deep sedation with two interventionalists in a fixed team each performingaround 150 TAVIs per year. Selection of valve size was based on pre-procedural CT measurements. Predilatation was performed at the discretion of the interventionalist. The deployment was performed according to best practice recommendations. The aortic annular plane was defined in the CT analysis and adapted during the procedure to reach a perfect perpendicular view. Fast pacing (100–160 bpm) was performed during valve deployment in 92.6% (n = 251) of the patients, whereas no fast pacing pacing was performed in 7.4% (n = 20) of the patients due to low systolic blood pressure.

Retrospective data acquisition included baseline demographic information, imaging data from transthoracic echocardiography (TTE) and cardiac computed tomography (CT), procedural and post-interventional information and at least a three months clinical and echocardiographic follow-up. CT images were transferred to a dedicated workstation for evaluation (3mensio Structural Heart^™^, Pie Medical Imaging BV, Maastricht, The Netherlands) and the device landing zone calcification score (DLZCS, visual 4-step, semi-quantitative method to determine severity of calcification load of the aortic valve and surrounding tissue) [17] was assessed. For all patients a cover index was calculated which was defined as ratio of 100 x ([prosthesis actual diameter at implantation depth—annulus diameter]/prosthesis actual diameter at implantation depth). Image acquisition was performed in accordance with standardized recommendations [18, 19]. Clinical study endpoints were obtained in accordance with the updated valve academic research consortium (VARC-2) [19] criteria.

**Fig 2:**
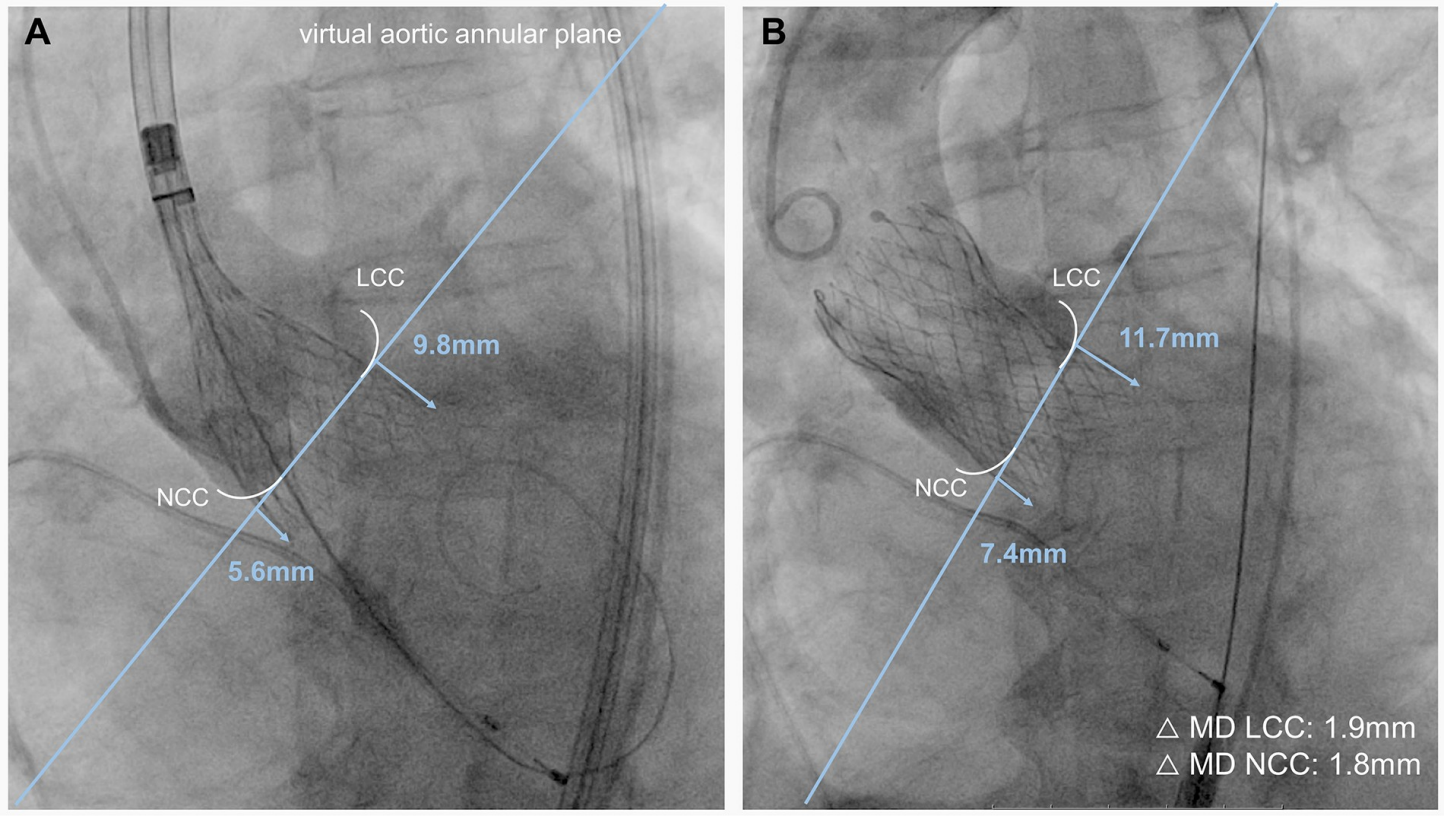
Exemplary illustration of micro-dislodgement during transcatheter aortic valve implantation. **(A)** Prosthesis in the final phase of valve deployment shortly before release. The implantation depth is measured form the virtual aortic annular plane to the interventricular end of the transcatheter heart valve. Distance measurements are performed in regard to the left coronary (LCC) and the non-coronary cusp (NCC). **(B)** Angiographic assessment of the final valve position. Distance measurements are once again performed. In this example micro-dislodgement of the transcatheter heart valve occurred in direction of the left ventricular outflow tract with a magnitude of 1.9mm in relation to the LCC and 1.8mm to the NCC, respectively.

MD was defined as the movement of the prosthesis of at least 1.5 mm from its initial position directly before the final release compared to the definite position after the release (exemplary illustration in Fig 2). Smaller valve deflections could not be reliably reproduced by different observers, which explains the chosen cut-off. The movement either took place in direction of the LVOT or towards the aorta. The implantation depth was determined angiographically in the perpendicular plane chosen for valve deployment. Distance measurements from the interventricular end of the prosthesis to the annular plane were performed afterwards, using the PACS system workstation SECTRA IDS7 (Sectra AB, Linköping, Sweden). To assess the intra- and interrater variability 50 patients were randomly chosen and the implantation depth was measured by two physicians, who were blinded to the results.

Statistical analysis was performed with SPSS (IBM). Continuous variables are expressed as means ± SD and compared using a Student`s t-test or Mann-Whitney U-test depending on variable distribution. Categorical variables were compared using chi-square testing or Fisher exact test. The interobserver variability was calculated with the intercorrelation coefficient (ICC) and can be interpreted as follows: >0.8 excellent agreement, 0.6–0.8 fair to good agreement, 0.4–0.6 moderate agreement and <0.4 no agreement. A p-value <0.05 was considered statistically significant. A multivariate regression analysis was performed to identify independent predictors for the occurrence of MD. Parameters with a p<0.1 in the univariate analysis were included in the multivariate regression analysis.

## Results

Our study population consisted of 258 patients who underwent transfemoral TAVI with the CoreValve or CoreValve Evolut R^™^ from 03/2015 to 03/2017 at the Heart Center Düsseldorf. MD was observed in 82 (31.8%) patients and the mean magnitude was 2.8 ± 2.2 mm in relation to the left coronary cusp (LCC) and 3.0 ± 2.1 mm to the non-coronary cusp (NCC). In the majority of cases, the prosthesis moved towards the aorta (n = 51, 62.2%) whereas in 37.8% a movement towards the LVOT was observed. Overall, devices were deeper deployed in the LVOT if MD occurred (distal prosthesis end to the NCC -MD vs. +MD: 5.2 ± 2.5 mm vs. 6.6 ± 3.3, p = 0.001 and distal prosthesis end to the LCC -MD vs. +MD: 6.4 ± 2.1 mm vs. 7.3 ± 3.0 mm, p = 0.025). The ICC for the distance measurements of the NCC was 0.923 and for the LCC 0.899, showing an excellent agreement. Pre-procedural CT data analysis revealed an annulus perimeter of 74.8 mm ± 6.9 in the -MD group and 74 mm ± 6.1 in the +MD group (p = 0.396). The most calcified cusp was the NCC in both groups (-MD vs. +MD: 40.9% vs. 31.7%; p = 0.200) and symmetric calcification distribution was more common in the +MD group (-MD vs. +MD: 27.3% vs. 40.2%; p = 0.037). DLZCS did not differ if mild and moderate calcification was compared to severe and massive calcification (DLZCS 3+4 -MD vs. +MD: 54.0% vs. 59.8%, p = 0.384). Additional CT-derived data can be found in Table 1.

**Table 1:** CT derived data.

DLZCS	-MD (n = 176)	+MD (n = 82)	p-value
1 (%)	31 (17.6)	23 (28.0)	0.055
2 (%)	64 (36.4)	26 (31.7)	0.465
3 (%)	55 (31.3)	20 (24.4)	0.258
4 (%)	26 (14.8)	13 (15.9)	0.821
**Most calcified cusp**			
Symmetric (%)	48 (27.3)	33 (40.2)	0.037
Non-coronary cusp (%)	72 (40.9)	26 (31.7)	0.200
Left coronary cusp (%)	34 (19.3)	10 (12.2)	0.156
Right coronary cusp (%)	22 (12.5)	13 (15.9)	0.463
Calcification load RCC (HU)	27.8 ± 26.8	358.0 ± 47.9	0.090
Calcification load NCC (HU)	386.8 ± 30.3	472.9 ± 60.2	0.156
Calcification load LCC (HU)	302.7 ± 28.6	372.9 ± 52.2	0.202
Calcification load total (HU)	3846.8 ± 331.4	4798.5 ± 628.3	0.143
Calcification load LVOT (HU)	287.9 ± 35.4	410.7 ± 65.1	0.073
Perimeter annulus (mm)	74.8 ± 6.9	74.0 ± 6.1	0.396
Area derived annulus (mm^2^)	439.6 ± 83.5	430.9 ± 70.5	0.532
Mean diameter annulus (mm)	23.2 ± 2.4	23.1 ± 2.0	0.532
Left ventricular outflow tract (cm^2^)	3.6 ± 0.8	3.7 ± 0.9	0.734
Sinotubular junction (mm)	27.3 ± 3.0	27.4 ± 3.5	0.679
Sinus of valsalva (mm)	31.6 ± 3.9	32.0 ± 4.6	0.491
Ascending aorta (mm)	32 ± 3.9	32 ± 4.3	0.340
Sinus height	27 ± 3.0	27 ± 3.5	0.486
Distance to the RCA (mm)	15 ± 3.2	15 ± 3.5	0.562
Distance to the LCA (mm)	13 ± 2.7	14 ± 2.9	0.066

DLZC: Device landing zone calcification

LCA: Left coronary artery

RCA: Right coronary artery

### Baseline characteristics

The mean age of the study population was 81 years ± 5.6 in the -MD group and 80 years ± 7.1 in the +MD group (p = 0.123); 68.6% of the patients were female. The patient cohort represents a real-world high-risk collective with a mean log EuroSCORE I of 28.8% ± 16.8 in the -MD group vs. 28.8% ± 15.3 in the +MD group (p = 0.997). Baseline characteristics did not differ between both groups. Echocardiographic assessment of the valve function showed a smaller mean aortic valve area (AVA) in the -MD group (-MD vs. +MD: 0.6 cm^2^ ± 0.6 vs. 0.7 cm^2^ ± 0.2, p = 0.014). Patients in the -MD group presented more often with a good left ventricular function (-MD vs. +MD: 92.6% vs. 80.4%, p = 0.004). Baseline demographic and echocardiographic data are shown in Table 2.

**Table 2:** Baseline demographic data and echocardiographic assessment.

	-MD (n = 176)	+MD (n = 82)	p-value
Age (years)	81 ± 5.6	80 ± 7.1	0.123
Body mass index	27.1 ± 5.5	26.8 ± 4.9	0.728
Log EuroSCORE (%)	28.8 ± 16.8	28.8 ± 15.3	0.997
Female, n (%)	122 (69.3)	55 (67.0)	0.781
Diabetes, n (%)	55 (31.3)	20 (24.4)	0.258
Coronary heart disease, n (%)	128 (72.7)	56 (68.3)	0.463
- Coronary artery bypass grafting, n (%)	18 (10.2)	6 (7.3)	0.453
Neurological disease, n (%)	21 (11.3)	9 (11.0)	0.823
COPD, n (%)	64 (36.4)	31 (37.8)	0.823
Atrial fibrillation, n (%)	60 (34.1)	31 (37.8)	0.561
Arterial hypertension, n (%)	161 (91.5)	73 (89.0)	0.528
Cerebrovascular disease, n (%)	45 (25.6)	14 (17.1)	0.130
Peripheral vascular disease, n (%)	42 (23.9)	17 (20.7)	0.577
Pulmonary Hypertension, n (%)	125 (71.0)	59 (72.0)	0.878
Dialysis, n (%)	9 (5.1)	3 (3.7)	0.605
GFR (ml/min/1.73cm^2^)	52.7 ± 21.7	56.6 ± 20.6	0.180
Pacemaker, n (%)	23 (13.1)	13 (15.9)	0.548
**Echocardiographic data**			
Aortic valve area (cm^2^)	0.6 ± 0.6	0.7 ± 0.2	0.014
Pmean (mmHg)	38.3 ± 16.6	37.9 ± 17.0	0.880
Pmax (mmHg)	60.9 ± 25.4	61.2 ± 25.1	0.934
Left ventricular function			
Good, n (%)	163 (92.6)	66 (80.4)	0.004
Moderate, n (%)	5 (2.8)	10 (12.2)	0.007
Poor, n (%)	8 (4.5)	6 (7.3)	0.383

COPD: Chronic obstructive pulmonary disease

EuroSCORE: European System for Cardiac Operative Risk Evaluation

GFR: Glomerular filtration rate

Pmax: Maximal pressure gradient

Pmean: Mean pressure gradient

### Procedural characteristics

The mean procedure duration (-MD vs. +MD: 109 min ± 31.6 vs. 104 min ± 27.8, p = 0.304) and fluoroscopy time (-MD vs. +MD: 22 min ± 11.1 vs. 22 min ± 9.7, p = 0.904) did not differ between groups. Contrast agent administration was higher in the +MD group (-MD vs. +MD: 123 ml ± 38.7 vs. 137 ml ± 52.1, p = 0.020). In 6.2% (n = 16) of the patients a CoreValve was implanted, whereas 93.8% (n = 242) received a CoreValve Evolut R. Postprocedural complications according to VARC-2 were similar in both groups. Further in-hospital data can be found in Table 3.

**Table 3:** In-hospital data.

	-MD (n = 176)	+MD (n = 82)	p-value
**Prosthesis size**			
26 mm (%)	59 (33.5)	28 (36.8)	0.141
29 mm (%)	106 (60.2)	47 (61.8)	0.658
31 mm (%)	10 (5.7)	6 (7.3)	0.612
34 mm (%)	1 (0.6)	2 (2.4)	0.238
**Implantation depth (mm)**			
- In relation to LCC	6.4 ± 2.1	7.3 ± 3.0	0.025
- In relation to NCC	5.2 ± 2.5	6.6 ± 3.3	0.001
**Micro-dislodgement (mm)**			
- In relation to LCC		2.8 ± 2.2	
- In relation to NCC		3.0 ± 2.1	
- Towards the aorta		51 (62.2%)	
- Towards the LV		31 (37.8%)	
Procedure duration (min)	109 ± 31.6	104 ± 27.8	0.304
Fluoroscopy time (min)	22 ± 11.1	22 ± 9.7	0.904
Contrast agent (ml)	123 ± 38.7	137 ± 52.1	0.020
Cover index	18 ± 6.1	18 ± 5.0	0.377
Aortic regurgitation index post	23.6 ± 7.9	25.6 ± 8.1	0.069
Pre-Dilatation (%)	139 (79.0)	56 (68.3)	0.063
Post-Dilatation (%)	0 (0)	2 (2.4)	0.100
Cardiopulmonary resuscitation (%)	3 (1.7)	1 (1.2)	1.0
Re-capture and re-sheath (%)	13 (7.4)	4 (4.9)	0.594
Intubation (%)	5 (2.4)	2 (2.4)	1.0
Mechanical circulatory support (%)	0 (0)	1 (1.2)	0.318
Stroke (%)	8 (4.5)	2 (2.4)	0.511
Coronary obstruction (%)	1 (0.6)	0 (0)	1.0
Acute kidney injury (%)	1 (0.6)	2 (2.4)	0.238
Bleeding			
- Minor (%)	13 (7.4)	1 (1.2)	0.043
- Major (%)	1 (0.6)	2 (2.4)	0.238
Vascular complications			
- Minor (%)	15 (8.5)	6 (7.3)	0.360
- Major (%)	0 (0)	1 (1.2)	0.318
Delivery catheter system failure (%)	8 (4.4)	6 (7.9)	0.360
Sepsis (%)	4 (2.2)	1 (1.2)	0.568
Pacemaker (%)	19 (10.8)	13 (15.9)	0.251
**Echocardiographic data**			
Pmean (mmHg)	7 ± 3.4	8 ± 3.9	0.326
Pmax (mmHg)	14 ± 6.2	15 ± 7.0	0.280
Aortic regurgitation post implantation			
None/trace (%)	150 (85.2)	71 (86.6)	0.977
Mild (%)	25 (14.2)	11 (13.4)	0.661
Moderate (%)	1 (0.6)	0 (0)	1.0
30-day mortality (%)	1 (0.3)	3 (3.7)	0.096

LCC: Left coronary cusp

NCC: Non-coronary cusp

Pmax: Maximal pressure gradient

Pmean: Mean pressure gradient

### Echocardiographic outcomes and follow-up

**Fig 3:**
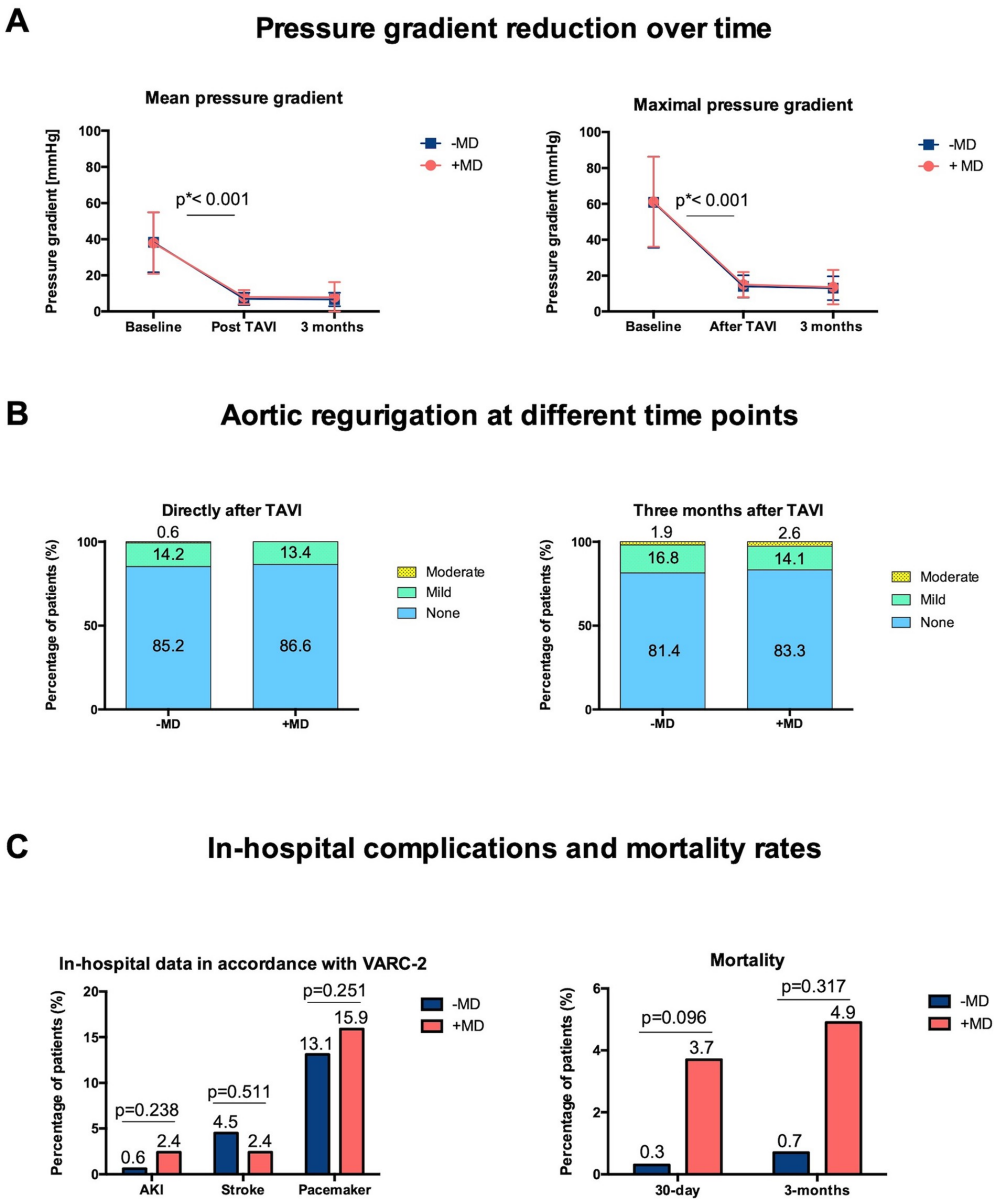
Echocardiographic outcome at different time points of the study, divided by groups. **(A)** Baseline mean and maximal pressure gradients could be effectively reduced after TAVI (baseline mean gradient -MD vs. +MD: 38.3 mmHg ± 16.6 vs. 37.9 mmHg ± 17.0 mmHg, p = 0.880 and after TAVI -MD vs. +MD: 7 mmHg ± 3.4 vs. 8 mmHg ± 3.9, p = 0.326) and showed consistency over a follow-up period of three months (-MD vs. +MD: 6.7 mmHg ± 3.7 vs. 7.9 mmHg ± 8.4, p = 0.168). **(B)** Directly after TAVI and at three months follow-up most of the patients presented with no aortic regurgitation (three months -MD vs. +MD: 81.4% vs. 83.3%, p = 0.711). Mild aortic regurgitation was observed in 16.8% of the -MD group and in 14.1% of the +MD (p = 0.597). Moderate aortic regurgitation occurred in 1.9% of all patients with no differences between groups (-MD vs. +MD: 1.9% vs. 2.6%, p = 0.662). **(C)** Procedure-associated complications were similar in both groups. 30-day (-MD vs. +MD: 0.3% vs. 3.7%, p = 0.096) as well as three-months mortality (-MD vs. +MD: 0.7% vs. 4.9%, p = 0.317) were comparable. LCC: Left coronary cusp. NCC: Non-coronary cusp. Pmax: Maximal pressure gradient. Pmean: Mean pressure gradient

The mean pressure gradient was effectively reduced in both groups (-MD vs. +MD: 7 mmHg ± 3.4 vs. 8 mmHg ± 3.9, p = 0.326) with consistency over a follow-up period of at least three months (-MD vs. +MD: 6.7 mmHg ± 3.7 vs.7.9 mmHg ± 8.4, p = 0.168 Fig 3A). Echocardiographic data at discharge showed a good result with no aortic regurgitation (AR) in most of the patients (-MD vs. +MD: 85.2% vs. 86.6%; p = 0.977, Fig 3B). At three months follow-up, the all-cause mortality did not differ between groups (-MD vs. +MD: 0.7% vs. 4.9%, p = 0.317, Fig 3C). Further follow-up data is displayed in Table 4.

**Table 4:** Three months follow-up.

	-MD (n = 161)	+MD (n = 78)	p-value
**Echocardiographic data**			
Pmean (mmHg)	6.7 ± 3.7	7.9 ± 8.4	0.168
Pmax (mmHg)	13.0 ± 6.6	13.6 ± 9.6	0.647
Vmax (m/s)	1.8 ± 0.7	1.9 ± 1.3	0.796
Aortic regurgitation			
None/trace (%)	131 (81.4)	65 (83.3)	0.711
Mild (%)	27 (16.8)	11 (14.1)	0.597
Moderate (%)	3 (1.9)	2 (2.6)	0.662
3-months mortality (%)	15 (0.7)	4 (4.9)	0.317

Pmax: Maximal pressure gradient

Pmean: Mean pressure gradient

### Risk factors for MD

To identify predictors for MD we performed a univariate regression analysis followed by a multivariate regression analysis. In this analysis, we found a lager AVA to be the only independent factor for the occurrence of MD (OR 5.3; 95% CI 1.1–24.9; p = 0.036, Table 5).

**Table 5:** Multivariate regression analysis.

	OR (95% CI)	p-value
Aortic valve area (cm^2^)	5.3 (1.1–24.9)	0.036
Left ventricular function (%)	1.9 (0.9–3.7)	0.073
AI index preprocedural	1.0 (0.9–1.1)	0.167
Calcification load RCC (HU)	1.0 (1.0–1.001)	0.083

## Discussion

MD during TAVI with self-expandable devices is an often observed phenomenon and predominantly occurs during the final phase of valve release. However, data focusing on this issue is scarce. Hence, we evaluated the incidence, potential risk factors and related clinical outcome in the case of MD occurrence.

Major findings of our study are: 1.) MD occurred in almost one third of patients (31.8%), 2.) a lager AVA could be identified as the only independent risk factor for the occurrence of MD 3.) MD did not influence clinical and hemodynamic outcome over a follow-up period of three months.

Over the recent years, new-generation valve prostheses for TAVI have been developed, aiming to improve procedural outcome and patient`s safety [20, 21]. In addition, the implantation technique and pre-procedural assessment have moved forward to ensure optimal prosthesis placement and fitting [22]. This lead to a significant reduction of valve embolization and other severe adverse events. Previous studies identified the calcification load and under-sizing as accountable factors for valve embolization [23]. In contrary to an obviously severe adverse event like valve embolization [24] [18], the present analysis focuses on MD of the prosthesis. Since an acute clinical deterioration does not occur in the case of MD, the study puts emphasis on the clinical and hemodynamic results during the hospital course and the follow-up period.

### Valve deployment technique

In contrast to the rapid deployment balloon-expandable Edwards SAPIEN devices, the CoreValve prostheses consist of a nitinol stent frame. This material allows martensitic transformation and has a shape memory which enables high radial strength [25]. Self-expandable valves are mainly implanted with fast ventricular pacing (100–160 beats per minute) to reduce cardiac output, lower the arterial pulse pressure and transvalvular pulsatile flow. In combination with best practice handling of the delivery catheter system, re-sheathing and re-capture capabilities and controlled gradual deployment, the valve is ought to be positioned stable and precisely at the region of interest [20]. Despite all the previously mentioned measures taken, MD during the final release occurs and is triggered by a summation of mechanical forces. The crimped valve in the delivery system with tension, torque forces and radial expansion impulses faces the moving anatomy which can lead to uncontrolled valve displacement during the final phase. Next to these interacting, unpredictable mechanical forces during the final phase of valve deployment, we tried to identify predictable parameters for the occurrence of MD.

### Potential anatomical and procedure-related risk factors for MD

CT analysis only revealed a larger distance of the left coronary artery to the virtual aortic annulus (-MD vs. +MD: 13.1 mm ± 2.5 vs. 14.0 mm ± 2.7, p = 0.015) and a different predominant cusp calcification pattern as distinguishable anatomical factors between groups. However, in multivariate regression analysis none of these factors maintained significant. We found a larger AVA to be the only independent risk factor for MD potentially resulting in less internal friction, smaller radial forces of the prosthesis against the leaflets and increased freedom of movement of the prosthesis during the implantation.

A recent study found the angle between the ascending aorta and the transcatheter heart valve at the point of recapture, pre-dilatation and less operator experience to be independent predictors for valve dislodgement towards the LCC during the final phase of release [26]. As we had a very experienced and stable team, performing TAVI since 2010, we excluded operator experience in our study. Within this context, the angle of the ascending aorta and the transcatheter heart valve at the point of recapture is a factor, which has to be attributed to operator experience. Strict adherence to best practice recommendations with release of any tension or pulling force before the final release is mandatory in our center and predominantly excludes the angle to be an influencing factor in our study. Nevertheless, the individual experience may have in impact on the procedural result [27].

In the context of valve migration, pre-dilatation is an often discussed topic [28, 29]. Pre-dilatation was performed in 79% of the patients in the–MD group and 68.3% of the patients in the +MD group and had no impact on the occurrence of MD or the hemodynamic result. This observation is supported by another group, who was able to show, that pre-dilatation did not have an impact on valve dislodgement in CoreValve patients [30].

### Clinical and echocardiographic outcome

Even though MD occurred in one third of the patients undergoing TAVI with a self-expandablevalve, it did not negatively affect clinical and hemodynamic short- and mid-term outcome. In patients with MD, significantly more contrast agent had been used during the procedure, which did not result in a higher incidence of renal failure. Since the valve movement was recognizable by the implanting team immediately, MD probably led to additional contrast application in order to verify whether the device position was acceptable to ensure good clinical results. Our study is the first one showing that MD did not have an impact on short and mid-term clinical outcome and echocardiographic findings: the mean valvular pressure gradient was effectively reduced and aortic regurgitation after valve implantation was comparable in both groups. In contrast to complete valve dislocation, MD does not negatively affect hemodynamic and clinical outcomes during the in-hospital stay and three months follow-up.

## Conclusion

MD occurs in almost one third (31.8%) of patients but does not have an impact on short- and midterm clinical outcomes. We could identify a less narrow AVA as a potential risk factor for MD which may lead to more mobility of the valve during the final release. Further predictors, such as anatomical risk configurations or calcification patterns could not be identified.

## Limitations

This is only a single center study with retrospective data acquisition. Further multi-center, prospective trials with higher patient numbers and longer follow-up durations are needed to create evidence-based knowledge in the field of MD.

## Supporting information

S1 File Data copy. (XLSX)

## Abbreviations

AS: Aortic stenosis
AVA: Aortic valve area
CT: Computed tomography
DLZCS: Device landing zone calcification score
EuroSCORE: European System for Cardiac Operative Risk Evaluation
LCC: Left coronary cusp
LVOT: Left ventricular outflow tract
MD: Micro-dislodgement
NCC: Non-coronary cusp
PVL: Paravalvular leakage
TAVI: Transcatheter aortic valve implantation
TTE: Transthoracic echocardiography
VARC: Valve academic research consortium
